# Calligraphic interdigitated capacitive sensors for green electronics

**DOI:** 10.1038/s41598-024-64461-2

**Published:** 2024-07-08

**Authors:** Abhay Singh Thakur, Vinit Srivatava, Hyeong Kwang Benno Park, Imen Kebaili, Imed Boukhris, Yun Hwan Joo, Tae Hyun Sung, Anuruddh Kumar, Rahul Vaish

**Affiliations:** 1 Indian Institute of Technology Mandi, Mandi, Himachal Pradesh 175005 India; 2 Department of Electrical Engineering, Hanyang University, Seoul, 04763 South Korea; 3 Department of Physics, Faculty of Science, King Khalid University, P.O. Box 9004, Abha, Saudi Arabia; 4 Center for Creative Convergence Education, Hanyang University, Seoul, 04763 South Korea

**Keywords:** Pencil, Wood, Interdigitated capacitive sensor, Arrow key functionality, Electrical and electronic engineering, Engineering, Materials science

## Abstract

This study presents a novel approach to fabricating interdigitated capacitive (IDC) touch sensors using graphite-based pencils on a wood substrate. The sensors were designed to detect touches and pressure variations, offering a cost-effective and environmentally friendly solution for sensor fabrication. The fabrication process involved abrasion of graphite pencils on a wooden substrate to create conductive traces, followed by the integration of interdigitated electrode structures. Capacitance variations resulting from touch interactions were investigated to calibrate sensor responses for tailored tasks. The sensitivity of the sensor was found to be 1.2 pF/kPa, highlighting its responsiveness to pressure variations. Additionally, the sensors were interfaced with an Arduino Uno microcontroller board to demonstrate practical applications, such as replicating arrow key functionality. Additionally, the sensors exhibit sensitivity to environmental factors, with the relative change in capacitance increasing from 0.1 to 0.65 as relative humidity ranges from 30 to 90%. Furthermore, variations in temperature from 30 to 60ºC result in a relative change in capacitance increasing to approximately 0.5. The results indicate the feasibility and versatility of using wood-based substrates and graphite-based pencils for fabricating IDC touch sensors, offering promising prospects for sustainable and accessible sensor technology.

## Introduction

The ongoing advancements in electronics are paving the way for a diverse array of application, ranging from implantable biomedical devices to soft robotics to sustainable sensors and energy-harvesting tools for everyday consumer electronics^1^. The evolution of electronic technologies has also greatly expanded the capabilities, with capacitive touch sensors along with foldable sensors receiving notable attention for their easy setup, efficiency, and reliability^2,3^. They cater to diverse applications like interaction, humidity, and proximity sensing. Interdigitated Capacitive (IDC) sensors have been prevalent since the 1970s^4^. These rapid technological progress drives increased electronic device utilization, resulting in increased e-waste production and environmental concerns. As more and more electronic gadgets are being used, the amount of electronic waste (e-waste) created is increasing^5^. This is leading to significant environmental challenges. Organic substrates add to electronic waste and struggle to break down while also struggling to hold onto sensing materials^6^. The growing interest in flexible, proximity, foldable, and wearable electronics is rooted in their versatile potential across sectors. Within this context, foldable sensors also play a crucial role^7^. Despite the flexibility inherent in organic substrates like PET(Polyethylene Terephthalate), PDMS(Polydimethylsiloxane), they pose challenges tied to electronic waste and^8,9^.

There is increasing interest in developing electronic devices using flexible, eco-friendly materials and innovative manufacturing methods to address concerns related to e-waste, and the limitations of current electronics. The inappropriate management of e-waste can yield numerous detrimental outcomes, impacting the environment, human health, and data security. Growing electronic waste, containing 20% plastic, poses health and environmental risks due to heavy metals and harmful additives^10^. Current techniques for sensor electronics involve costly, toxic materials and environmentally harmful processes. These methods, including lithography and printing, yield non-customizable sensors with limited applications^11,12^.

To address these issues, a more effective, consistent, and environmentally friendly approach is needed. The focus is now shifting towards using degradable and flexible substrate materials to optimize the sensor performance. To tackle this concern, exploring eco-friendly, paper and wood have emerged as an alternative to non-biodegradable based versions. Paper offers flexible, lightweight, and cost-effective integration of electronic components^13^. Paper has diverse applications including wearables^14,15^ and touch displays^16^. The affordability of cellulose-based paper has led to its widespread adoption in packaging, electronics like circuit boards, solar cells, and sensors^17^,^18^,^19^. Paper electronics, though promising, come with inherent limitations. These include durability concerns due to paper's susceptibility to degradation from moisture and mechanical stress. Additionally, paper's electrical and mechanical properties may not match those of conventional substrates, leading to performance limitations. Environmental sensitivity, manufacturing complexity, and limited integration capabilities further hinder the widespread adoption of paper-based electronic devices.

Wood, known for being widely available, affordable, and friendly to the environment, has caught attention in eco-friendly electronics and energy fields. Wood has emerged as a versatile substitute for various non-biodegradable materials, owing to its abundant availability and diverse properties. It has been harnessed for a multitude of applications, including the fabrication of circuit boards^20^, sensors^21^ and energy storage devices such as batteries^22^ and super capacitors^23^. With a wide array of wood types boasting distinct characteristics such as robustness, porosity, and flexibility, the scope of applications for wood is extensive. These properties enable the creation of both robust and flexible sensors^24^, catering to diverse needs. Additionally, the porous nature of wood is leveraged for energy storage applications^25,26^, enhancing its utility further. Fabricating electrodes on wood necessitates the use of various techniques, including printing^27^, laser carbonization^28^, conductive inks^29^, and carbon nanotubes^30^, to ensure efficient integration of electronic components. This amalgamation of wood's inherent properties and advanced fabrication methods underscores its potential in advancing electronic device technology.

In this study, we present a calligraphic and cost-effective method for fabricating interdigitated capacitive touch sensors by abrasion of graphitic pencils on wooden substrates. The impact of touch on sensor capacitance values was investigated, providing insight into calibration possibilities for tailored tasks. For practical application i.e. to replicate arrow key functionality, these sensors were interfaced with a microcontroller board namely Arduino Uno. In addition, the sensing performance of the touch sensors was systematically investigated at room temperature and the effect of the external environment on it.

## Materials and characterization

In the development of biodegradable and eco-friendly interdigitated capacitive sensors, a range of pencil grades, including HB, 2B, 4B, 6B, and 8B, were employed as the core material. These pencils, with varying graphite content, played a pivotal role as the electrode material for the sensors. Pine wood, selected for its suitability in inter-resisted applications, served as the substrate for sensor fabrication. Characterization of these sensors, particularly assessing their touch sensitivity, was accomplished using the Keithley SCS4200 instrument, renowned for its precision in measuring capacitance changes upon touch stimuli. Additionally, the integration of the Arduino microcontroller facilitates the precise quantification of capacitance changes, enhancing its applicability in electronic devices. Notably, capacitance measurements were conducted using the Keithley SCS4200 within a controlled environment—a Class 100 cleanroom facility—to ensure the highest level of data accuracy.

### Sensor fabrication

**Figure 1:**
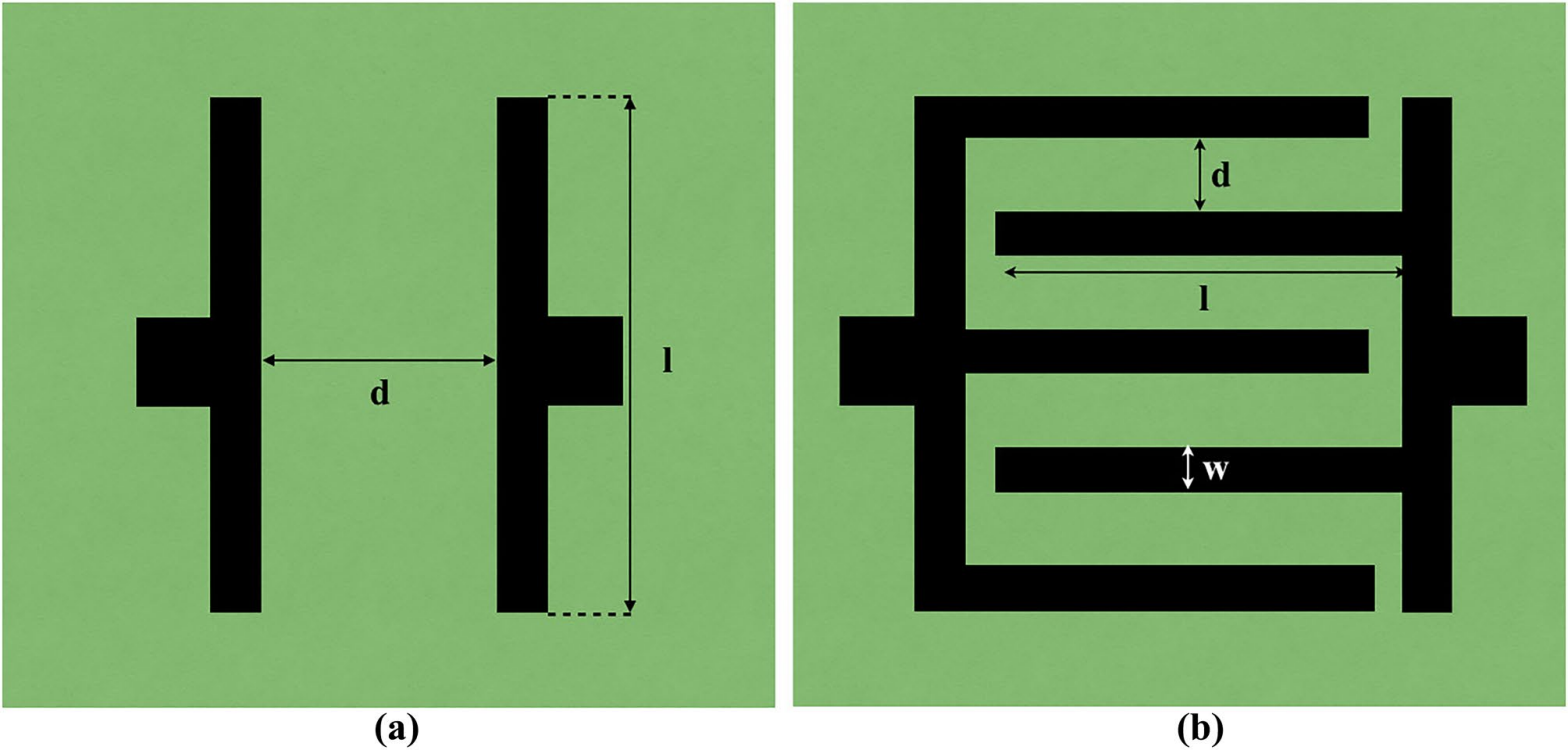
Schematic drawing (**a**) Interdigitated capacitive sensor with no Fingers. (**b**) Interdigitated capacitive sensor with 5 Fingers.

In the fabrication of interdigitated capacitive sensors, a wooden substrate served as the base, and graphite of grade 8B pencil was carefully deposited through abrasion. Precision was paramount, achieved through a regulated number of traces, uniform speed, and consistent pressure application. Although minor resistance variations were inevitable due to manual work, close uniformity was maintained. Figure 1a and b, illustrate selected key design. Figure 1a showcases the interdigitated capacitive sensors with zero fingers, featuring a parallel electrode configuration with no interlocking elements. In contrast, Fig. 1b represents sensors with five fingers. This design choice hinged on scientific reasoning—the more fingers, the greater the overall capacitance. However, this study predominantly centers on precise touch detection, akin to keyboard arrow keys, necessitating reduced capacitance. Thus, the choice leaned towards Fig. 1A, eliminating proximity sensing to focus solely on touch-based interactions. This decision is grounded in established principles, as the number of fingers increases, the effective area of a capacitor increases, leading to higher capacitance and subsequently, and greater sensitivity. However, since our primary focus is touch-based sensing, it opts for the zero-finger interdigitated capacitive sensors.

The functioning of our interdigitated capacitive sensor is rooted in the principles of capacitance variation, triggered by alterations in the dielectric properties within the sensor's electric field. As a human finger approaches or makes contact with the sensor's surface, a combination of factors comes into play. The first key factor is the conductive nature of the human finger. As the finger draws near, it essentially becomes an integral part of the electrical circuit, interacting with the electric field generated between the sensor's electrodes. The second crucial factor is the alteration of dielectric properties. Normally, the region between the electrodes contains air, which has a distinct dielectric constant. However, as the human finger intrudes into this electric field, it changes the dielectric properties, leading to modifications in the electric field lines and subsequently, capacitance. In practical terms, this interaction leads to a measurable change in capacitance. The total capacitance within the interdigitated capacitor is comprised of two primary components: the initial capacitance (${C}_{0}$) and the capacitance influenced by the touch (${C}_{p}$). When these two capacitances were combined, they form the overall effective capacitance of the sensor. The measurement of these capacitance changes was facilitated by advanced instrumentation, including the Keithley 4200SCS. This instrument allowed us to apply a 25 mV AC signal, enabling precise capacitance measurements across various frequencies. These measurements were instrumental in ensuring the operational stability of our constructed sensor. To prepare the sensor samples for measurement, a pre-soaking process with a 5 V DC current was conducted. This step was crucial for reducing the tunneling effect and polarizing the wood substrate, which induced surface charge and enhanced our ability to detect substantial changes in capacitance.

**Figure 2:**
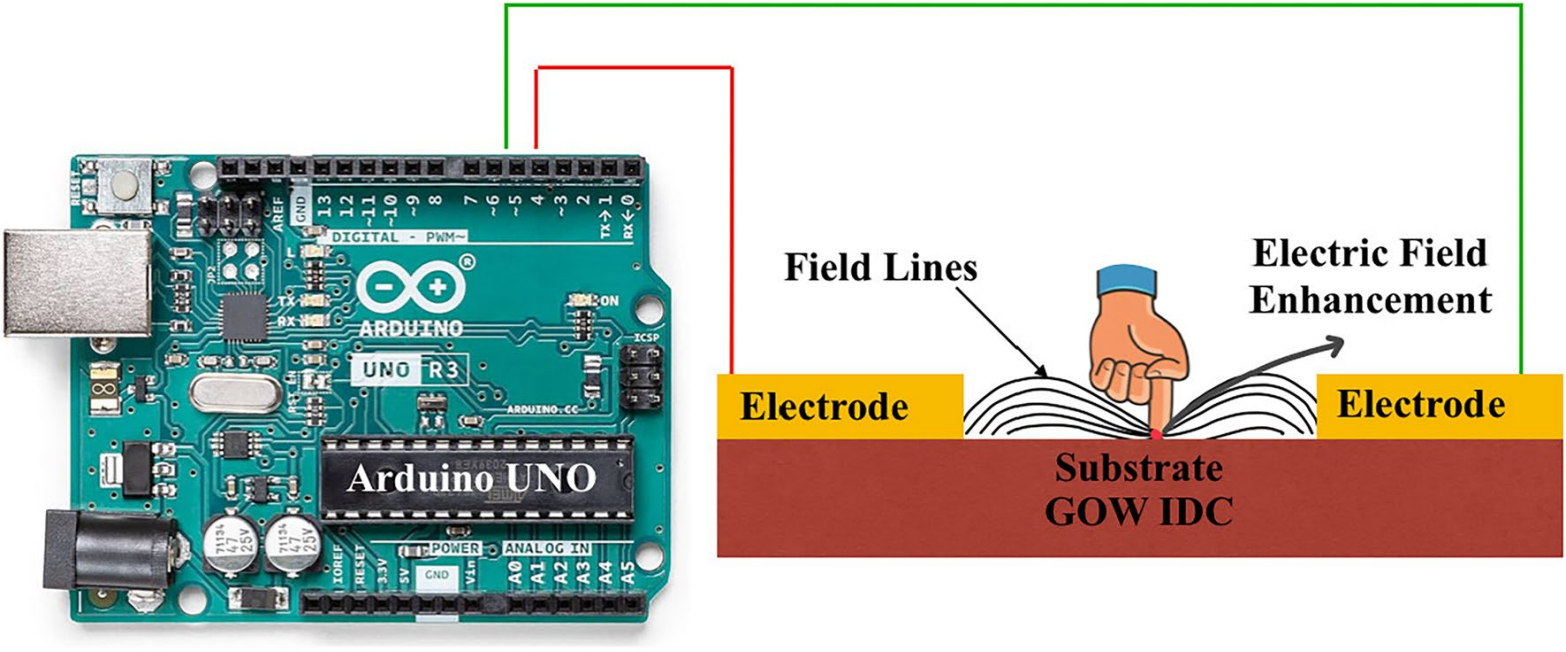
Visual representation of electric field lines interacting between finger and the IDC sensor.

As Fig. 2 provides a visual representation of our measurement process. In this setup, an Arduino microcontroller is connected to the sensor. As a hand approaches the sensor, disturbances in the electric field are detected, and changes in capacitance are accurately measured.

## Results and discussion

In this research, a significant effort was dedicated to explore different designs of IDC sensors suitable for touch applications. The primary focus was on evaluating the performance of wood substrates in conjunction with IDC sensors. Additionally, pencils with varying graphite content, readily available in the market, were assessed as potential candidates for fabricating IDC electrodes on wood substrates. Graphite's conductive properties made it an appealing choice for electrode fabrication on wood. The extensive goal of this research was to replicate the functionality of computer arrow keys using simple handmade IDC sensors on wood substrates.

The objective was to compare and identify the most suitable combinations of materials and designs. Multiple wood samples were subjected to thorough electrical property analyses. Furthermore, a meticulous manual technique was employed to create pencil drawings on the wood surfaces. This approach ensured precision and consistency in electrode fabrication. Maintaining uniformity in graphite deposition and precise control over electrode dimensions were vital aspects of the fabrication process. Fixed pressure and frequency were employed during the fabrication process to achieve these objectives.

Through this comprehensive investigation of wood substrates, the research aimed to provide valuable insights into the behaviour of IDC sensors and their compatibility with specific materials. This study not only shed light on the electrical performance of IDC sensors on wood substrates but also contributed to the development of efficient and reliable IDC sensors for use on wooden surfaces. IDC sensors hold promising potential for various eco-friendly applications, particularly in industries where wood-based substrates are commonly employed for touch-sensitive applications. Theoretical capacitance values for interdigitated capacitive sensors can be calculated using Eq. (1)^31^, which incorporates parameters such as figure width (a), electrode thickness (t), dielectric constants in free space (ε_0_) which is 8.8542 × 10 − 12 F/m, substrate (ε_r_), and dielectric film (ε_k_). The equation utilizes the complete elliptic integral of the first kind (K) to derive these values.

1 $${C}_{uc}={\in }_{0}\left({\in }_{r}+{\in }_{k}\right)\frac{K\left(\sqrt{1-{(a/b)}^{2}}\right)}{K(a/b)}+2{\in }_{0}{\in }_{k}\frac{t}{a} $$

**Figure 3:**
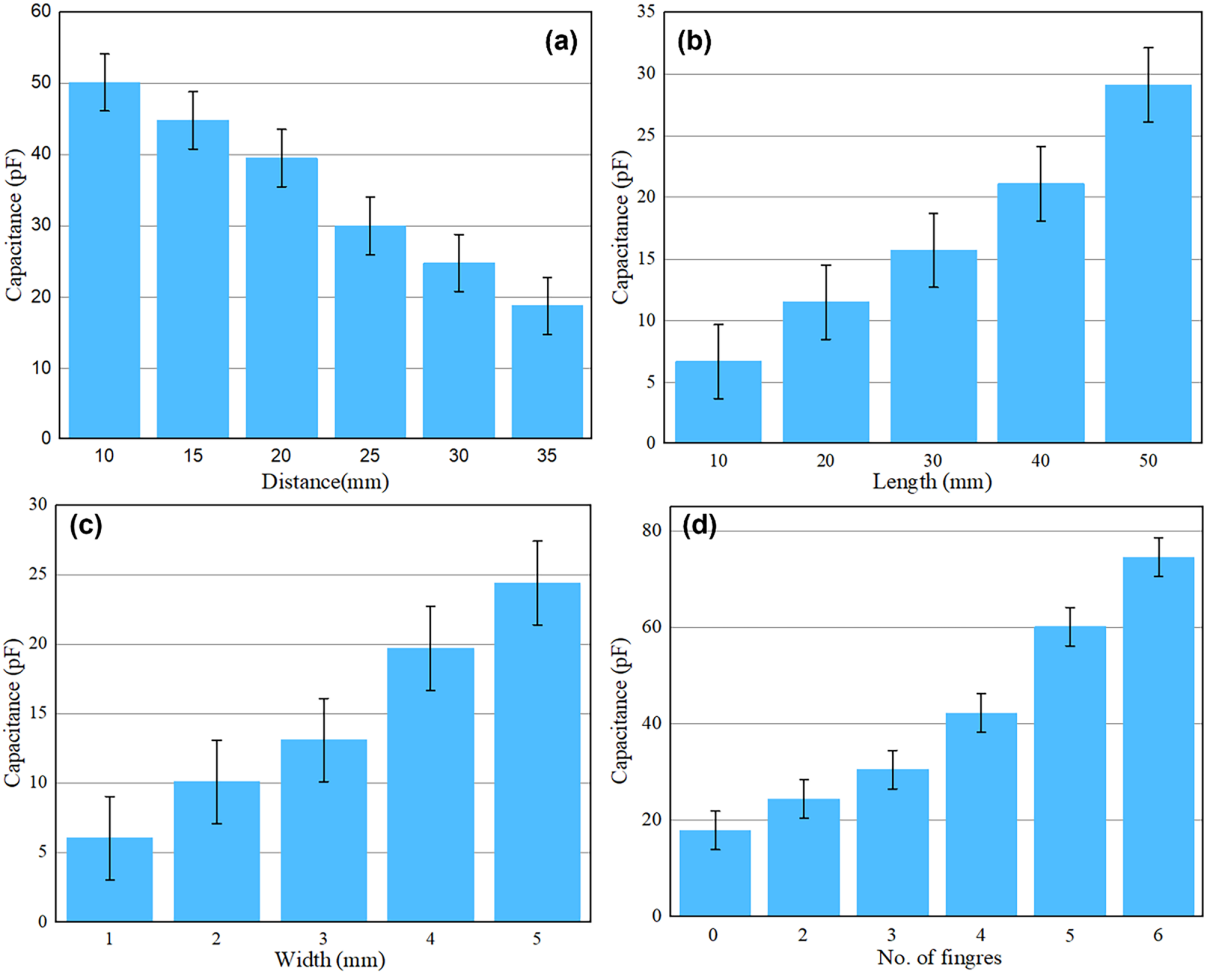
Parametric study of Idc sensors: (**a**) Capacitance vs distance, (**b**) Capacitance vs electrode length, (**c**) Capacitance vs electrode width and (**d**) Capacitance vs number of fingers.

In this section, the parametric study on the sensor has been performed, examining how changes in electrode distance, length, width, and the number of fingers affect capacitance. This study involved altering one factor at a time while keeping the others constant. The significance of this study lies in its ability to evaluate the influence of these factors on the sensor's performance. Figure 3a Capacitance vs. Distance—Capacitance decreases with distance primarily because of the diminishing electric field strength between the sensor's electrodes and the interacting object. As the distance grows, the electric field weakens, resulting in a lower capacitance value. Figure 3b Capacitance vs. Length—When the length of the electrode increases, so does the capacitance. This occurs because a longer electrode offers a larger surface area for the electric field to interact with external objects. Consequently, this amplifies the capacitance. Figure 3c Capacitance vs. Width—Widening the electrode enhances capacitance because it provides a broader area for the electric field to engage with nearby objects. The increased surface area intensifies the capacitance measurement. Figure 3d Capacitance vs. Number of Fingers—As the number of fingers interacting with the sensor rises, the effective area of the capacitor increases. This heightened effective area amplifies the sensor's ability to detect changes in capacitance, resulting in an increase as the number of fingers grows.

This investigation centres around exploring the intricate relationship between touch strength and capacitance within the realm of interdigitated capacitive sensors fabricated on wood substrates, employing pencil traces for electrodes. Our experimental methodology was designed to systematically manipulate touch strength while concurrently measuring the corresponding changes in capacitance. The ensuing results not only shed light on the nuanced behaviour of this touch sensor system but also hold the promise of far-reaching implications for the development of responsive touch sensors.

**Figure 4:**
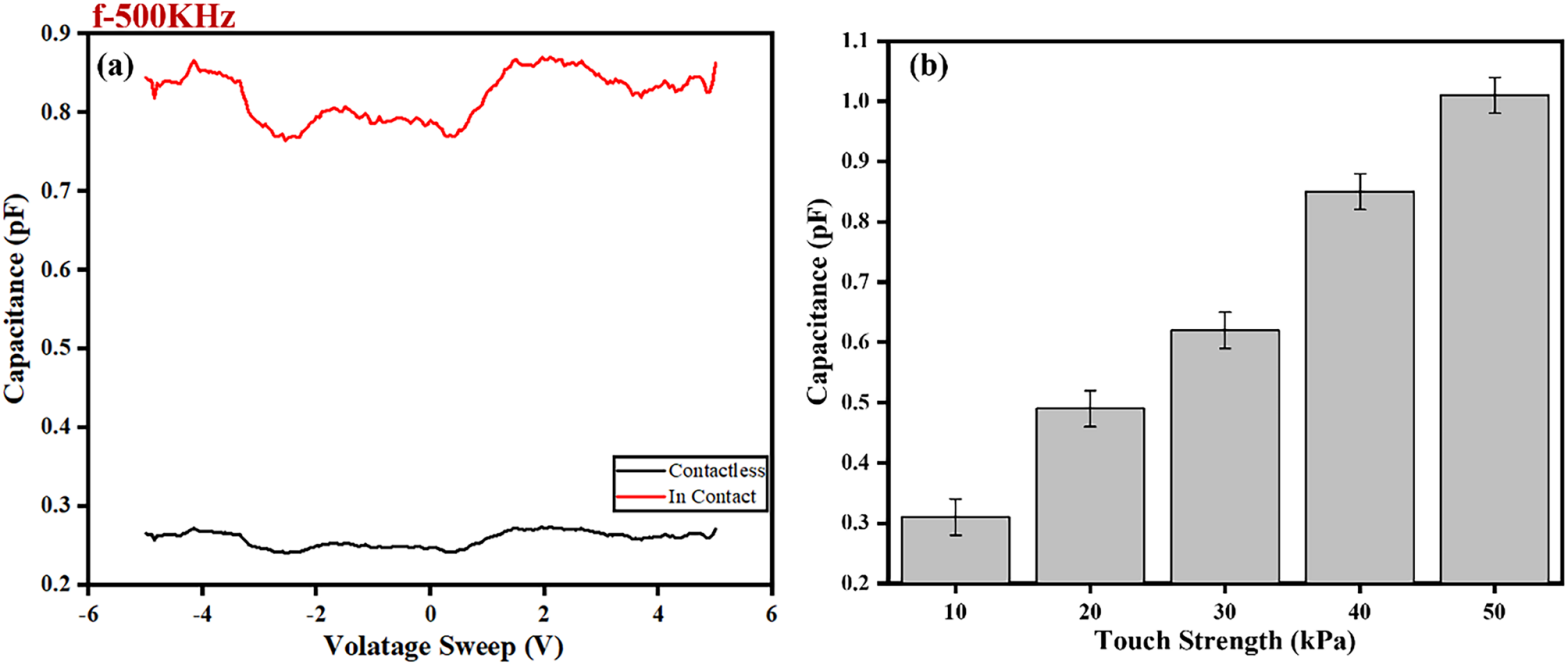
(**a**) Capacitance changes with voltage sweep, increasing upon touch and (**b**) Capacitance change with touch strength.

Figure 4a, presents data obtained using the Keithley SCS4200 instrument, demonstrating the relationship between voltage sweep and initial capacitance. Initially, the sensor's capacitance was measured within the range of − 5 to 5 V. In its untouched state, the sensor exhibited an initial capacitance of approximately 0.25 pF. However, upon physical contact with the sensor, a notable increase in capacitance was observed, with values rising to approximately 0.85 pF. This dynamic shift illustrates how the act of touching the sensor results in a significant change in capacitance, showcasing its responsiveness to tactile input.

Figure 4b, delves into the correlation between touch strength and capacitance. In this experiment, varying levels of pressure is applied to the sensor using hand. The applied force ranged from 10 to 50 kPa, effectively simulating different touch strengths. A commercially available thin film pressure sensor, specifically the SF 45–65, was utilized to measure the pressure exerted by the hand on the sensors. In interdigitated capacitors (IDCs), capacitance is primarily determined by the overlapping area between the fingers of the interdigitated electrodes. An increase in pressure can lead to a larger surface area covered by the hand, resulting in more of the sensor being in contact. The resulting data clearly indicated that as touch strength increased, the capacitance of the interdigitated capacitive sensor exhibited a consistent and proportional rise. This suggests that the sensor's sensitivity is 1.4 pF per kPa within its sensing range. Although the experimental setup targeted pressures up to 50 kPa, initial evaluations hint at the possibility of extending the range to 80 to 100 kPa. These observations highlights the sensor's sensitivity to variations in touch strength, providing valuable insights into its potential applications and the fine-grained touch interactions it can detect.

**Figure 5:**
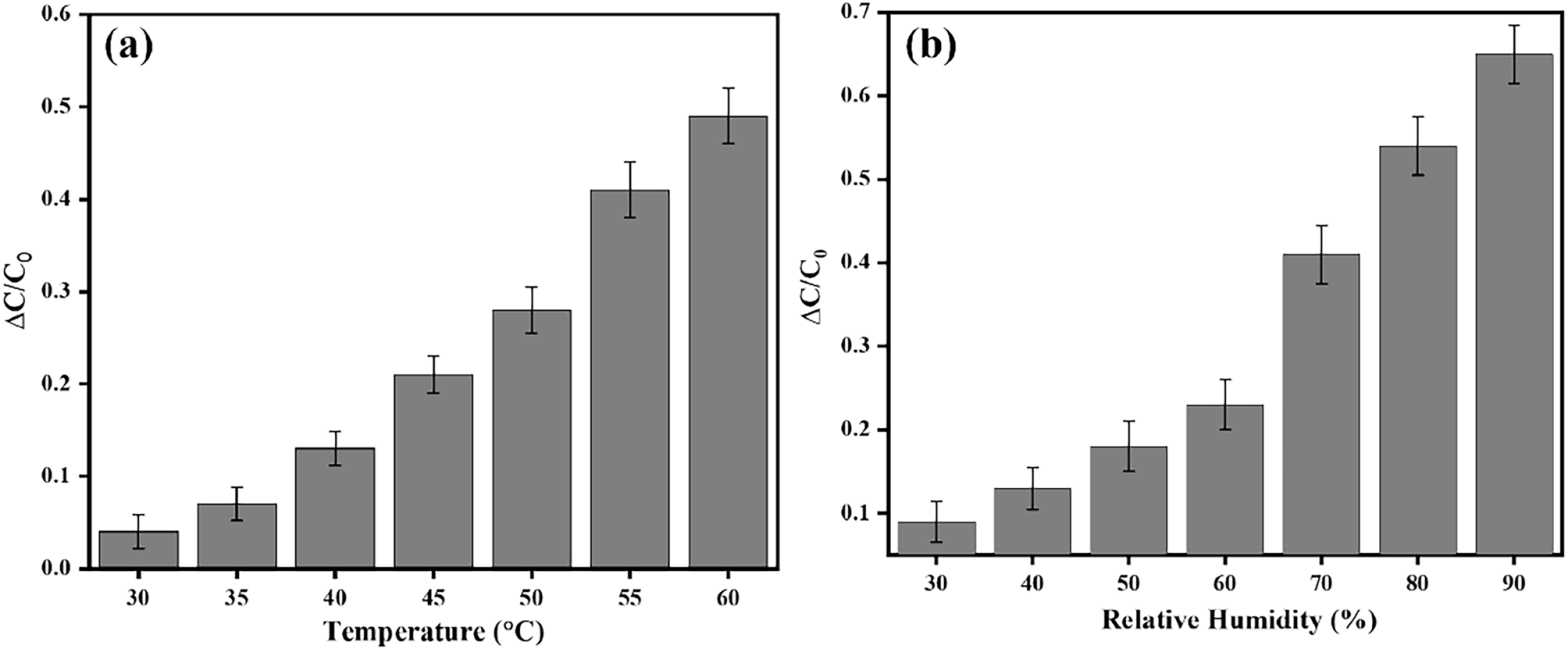
(**a**) Relative change in capacitance vs Relative humidity and (**b**) Relative change in capacitance vs Temperature.

The functioning of Inter-digitated capacitive sensors (IDC) on wooden substrates is significantly influenced by environmental parameters, given their practical applications. Among these factors, humidity and temperature stand out as key determinants affecting IDC sensor capacitance. A study was conducted to explore the impact of environmental conditions on these sensors. The graph in Fig. 5a depicts the relationship between relative humidity levels and relative capacitance changes. As relative humidity increases from 30 to 90%, capacitance changes range from approximately 0.1 to 0.65, indicating a non-linear trend. Specifically, a steep increase in capacitance change is observed as humidity levels surpass 60%. Similarly Fig. 5B illustrates the variation of capacitance concerning temperature. As temperatures elevate from 30 to 60 degrees Celsius, there is a discernible increase in capacitance values. Specifically, the relative change in capacitance ranges from 0.05 to approximately 0.5. This study underscores the substantial influence of environmental factors on IDC sensor performance, highlighting the necessity of considering such variables in the development and optimization of IDC-based systems and applications.

**Figure 6:**
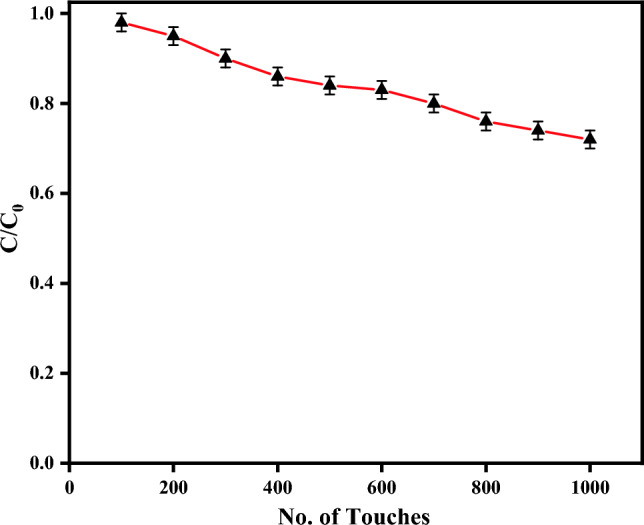
Effect on capacitance with number of touches.

The study investigates the impact of repeated touches on the capacitance of Inter Digitated capacitive sensors constructed through graphite abrasion on wooden substrates, designed for practical applications where frequent touching is expected. Figure 6A illustrates the relationship between the number of touches and the corresponding change in capacitance. As the number of touches escalates from 0 to 1000, the change in capacitance undergoes a transition from 1 to 0.75. While this change is not notably significant, it nonetheless suggests potential alterations in sensor properties, including sensitivity and other performance factors. This phenomenon arises from the gradual removal of graphite from the electrodes upon each touch, leading to incremental changes in capacitance over time. To address this issue, the application of a thin non-conducting film to shield the sensor can effectively prevent the erosion of graphite electrodes during touch interactions, thereby preserving sensor performance and consistency.

In the preceding sections of this research paper, the behaviour of inter-digitated capacitive sensors with zero fingers has been extensively explored, uncovering their remarkable ability to detect changes in capacitance when touched or subjected to pressure. Building upon these fundamental findings, now progressing to the practical application phase. Our aim is to harness this capacitive sensitivity to create a tangible sensor system with real-world utility. To accomplish this, versatile Arduino UNO microcontroller is used, which plays a pivotal role in measuring capacitance values and facilitating sensor actuation. The ultimate objective is to replicate the functionality of keyboard arrow keys, providing a tangible example of how the uncovered principles can be translated into a functional, touch-based input system. This article draws upon the insights gained from our prior research to bridge the gap between theoretical understanding and practical implementation, opening the door to a range of potential applications for inter-digitated capacitive sensors with zero fingers.

**Figure 7:**
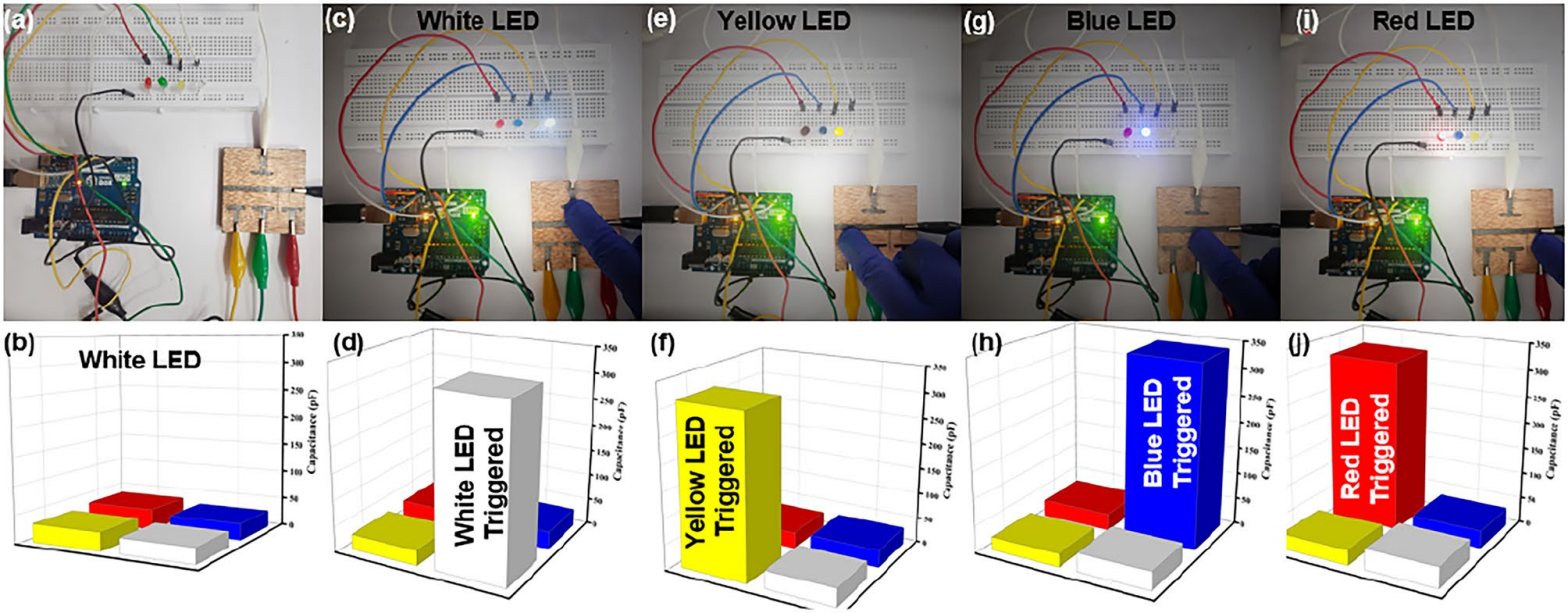
Real-world functionality of sensor system: (**a**) Sensors and actuators connected via Arduino Uno, (**b**) 3D bar graph displays initial capacitance values, (**c**) Sensor 1 touch activates white LED, (**d**) Peak in graph validates sensor 1 touch, (**e**) Sensor 2 touch illuminates yellow LED, (**f**) Peak confirms sensor 2 touch, (**g**) Sensor 3 touch activates blue LED, (**h**) Peak reflects increased capacitance for sensor 3, (**i**) Sensor 4 touch actuates red LED and (**j**) Red peak indicates increased capacitance for sensor 4.

To translate the theoretical insights from our research into practical real-world applications, Four sensors are fabricated on a hardwood substrate and precisely identified as Sensors 1, 2, 3, and 4. The sensors were colour coded to according to their actuators i.e. LED, Sensor 1 is connected to a white LED, Sensor 2 to a yellow LED, Sensor 3 to a blue LED, and Sensor 4 to a red LED. Figure 7a depicts the interconnection of the sensors with actuators through the Arduino Uno microcontroller. Figure 7b presents a 3D bar graph providing a visual representation of the initial capacitance values detected by these sensors through the microcontroller. In Fig. 7c, the sensor 1 registers a touch, leading to the activation of the white LED. This is represented by the distinctive peak in the white 3D bar graph in Fig. 7d, as it surpasses the predefined threshold and triggers the LED. Similarly, in Fig. 7e, sensor 2 responds to touch, causing the yellow LED to illuminate. Figure 7f showcases a peak in the yellow bar of the 3D bar graph, indicating the increase in capacitance for sensor 2. In Fig. 7g, sensor 3 is touched, leading to the activation of the blue LED. The corresponding peak in the blue bar of the 3D bar graph is visible in Fig. 7h. Lastly, Fig. 7i shows that sensor 4 has been touched, resulting in the actuation of the red LED. In Fig. 7j, a prominent red peak in the 3D bar graph signifies the increase in capacitance for sensor 4. It is important to highlight that when one sensor is pressed, any minute changes in the capacitance of other sensors remain well below the predefined threshold value. This demonstrates the specificity and precision of our sensor system in responding to individual touch inputs.

Figure 5, shows the sensor's operation with single presses. However, in real-world scenarios, multiple touches might occur simultaneously. To demonstrate the sensor's ability to handle multiple touches, some experiments were performed which are shown in Fig. 6.

**Figure 8:**
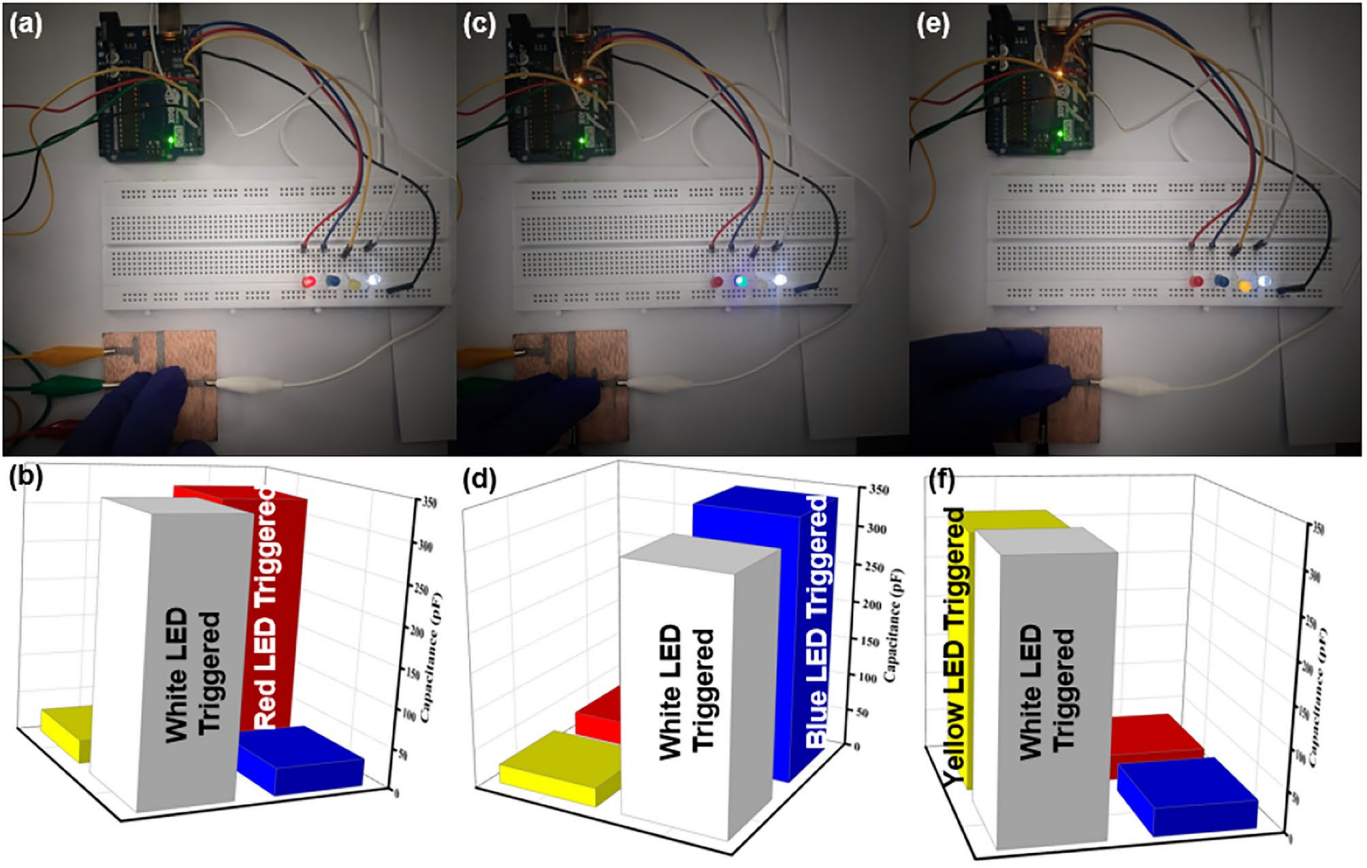
Multi-touch sensor detection: (**a**) Simultaneous pressing of sensor 1 and sensor 4 activates white and red LEDs, (**b**) Peaks confirm successful detection of multiple touches surpassing threshold, (**c**) Touching sensor 1 and sensor 3 illuminates white and blue LEDs, (**d**) Peaks in white and blue bar graphs confirm multi-touch detection, (**e**) Pressing sensor 1 and sensor 2 activates red and yellow LEDs and (**f**) Peaks in bar graph confirm touch detection on sensor 1 and sensor 2.

In Fig. 8a, two sensors, Sensor 1 and Sensor 4, were pressed, resulting in the activation of the white and red LEDs. Figure 8b displays the corresponding white and red bar graphs reaching their peaks, indicating the detection of multiple touches as they crossed the threshold. Figure 8c illustrates Sensor 1 and Sensor 3 being touched, leading to the activation of the white and blue LEDs. The peaks in the white and blue bar graphs in Fig. 8d confirm this multi-touch detection, while the other two sensors show minimal changes. In Fig. 8e, Sensor 1 and Sensor 2 were pressed, causing the red and yellow LEDs to activate. Similarly, in Fig. 8f, demonstrates that the white and yellow 3D bar graphs have reached their peaks, indicating an increase in capacitance in Sensor 1 and Sensor 2. These experiments demonstrate the robust performance of our sensor system, capable of accurately detecting multiple simultaneous touches.

This section deals with the deployment and demonstration of our interdigitated capacitive sensor system, which serves as the Arrow keys of the keyboard. The sensors fabricated in the above sections will be integrated with a computer system to replicate the functionality of traditional arrow keys. These sensors will enable functions such as increasing and decreasing volume, as well as forwarding and rewinding video playback.

**Figure 9:**
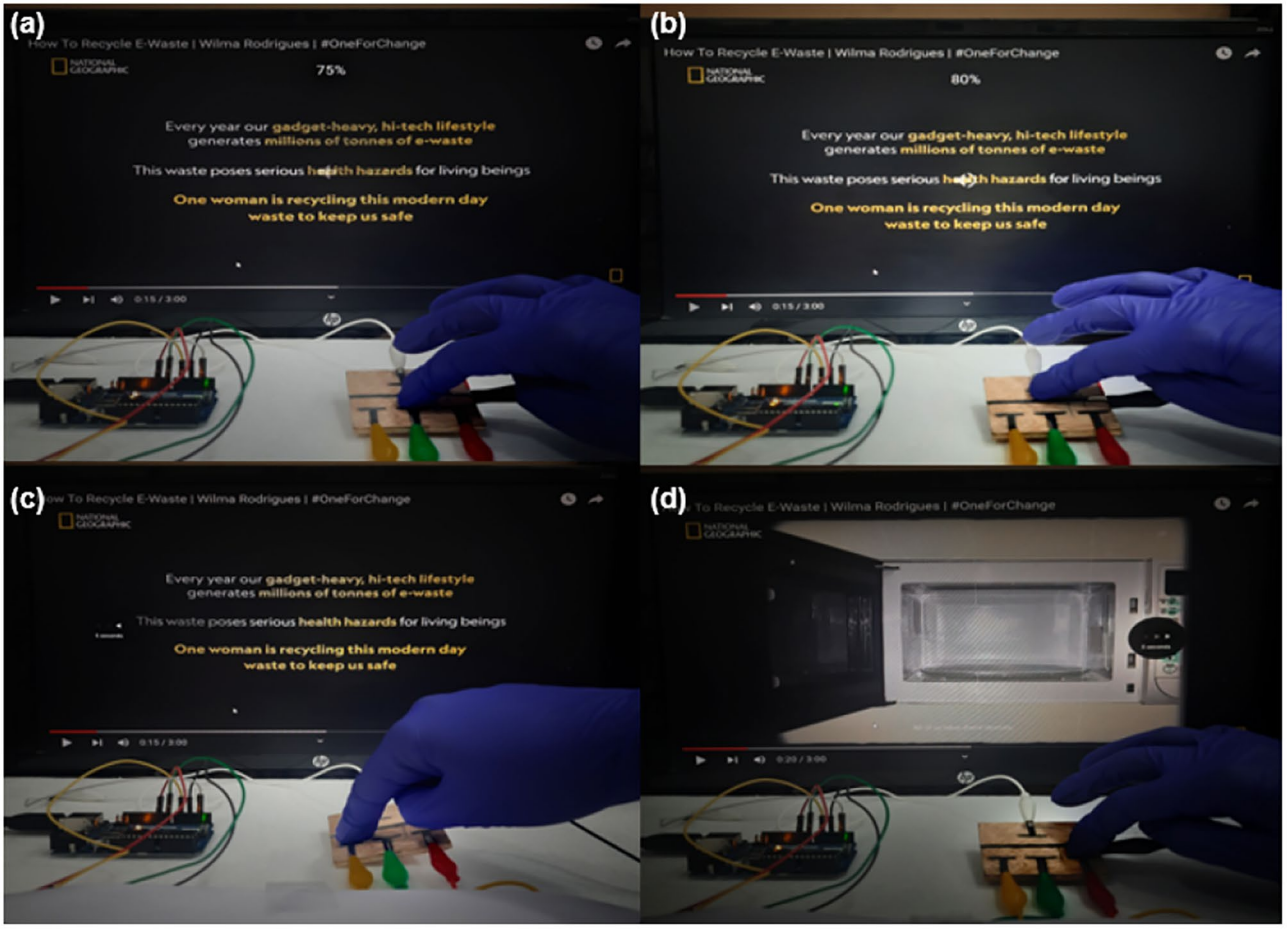
Replicating arrow key functionality: (**a**) Down key press decreases system volume, (**b**) Up key press increases system volume, (**c**) Left key press rewinds video by 5 s and (**d**) Right key press fast forwards by 5 s.

This section demonstrate the integration of the sensor system with a computer to replicate the functionality of arrow keys. Each sensor is assigned a specific function, with Sensor 1 acting as the up key, Sensor 2 as the left key, Sensor 3 as the down key, and Sensor 4 as the right key, effectively simulating the arrow key functions. In Fig. 9a, the down key press results in a volume decrease in the system. Conversely, Fig. 9b showcases the up key press, which leads to an increase in system volume. Figure 9c demonstrates the left key press, which rewinds videos by 5 s, while Fig. 9d illustrates the right key press, enabling fast forward by 5 s. The video has been uploaded as supplementary data. The successful integration of the sensor with the computer exemplifies the wide-ranging applications of this device beyond replicating arrow keys. It has the potential for various touch-based functions and utilizes eco-friendly materials, making it a promising technology for advancing touch-sensitive applications in an environmentally conscious manner.

## Conclusion

In conclusion, this study highlights the feasibility and potential of fabricating interdigitated capacitive touch sensors using graphite-based pencils on wood substrates. The investigations conducted throughout this study have revealed the sensors' effectiveness in detecting touch and pressure variations, with a measured sensitivity of 1.2 pF/kPa and minimal degradation in capacitance values even after 1000 touches. By systematically manipulating touch strength and the number of fingers involved, the dynamic nature of these sensors has been unveiled, showcasing their adaptability to real-world scenarios. The successful replication of arrow keys on a wood substrate and their seamless interaction with a computer system not only demonstrates their versatility but also hints at the eco-friendly touch-based interfaces of the future. Moreover, envisioning the prospect of keyless keyboards and the reduction of mechanical components in electronic devices could revolutionize our interaction with technology, making it more sustainable and efficient. Overall, this research underscores the promising prospects of Interdigitated Capacitive Sensors on wood substrates, offering a greener alternative for touch-based applications. As society increasingly values eco-friendly technologies, IDC sensors emerge as a remarkable contribution. This research propels us toward a brighter and greener technological future, where innovation aligns seamlessly with environmental responsibility.

### Supplementary Information

Supplementary Data.

## Data Availability

The datasets used and/or analysed during the current study available from the corresponding author on reasonable request.
